# Risk factor analysis and spatiotemporal CART model of cryptosporidiosis in Queensland, Australia

**DOI:** 10.1186/1471-2334-10-311

**Published:** 2010-10-28

**Authors:** Wenbiao Hu, Kerrie Mengersen, Shilu Tong

**Affiliations:** 1 School of Mathematical Sciences, Queensland University of Technology, Brisbane, Australia; 2 School of Population Health, University of Queensland, Brisbane, Australia; 3 School of Public Health, Queensland University of Technology, Brisbane, Australia

## Abstract

**Background:**

It remains unclear whether it is possible to develop a spatiotemporal epidemic prediction model for cryptosporidiosis disease. This paper examined the impact of social economic and weather factors on cryptosporidiosis and explored the possibility of developing such a model using social economic and weather data in Queensland, Australia.

**Methods:**

Data on weather variables, notified cryptosporidiosis cases and social economic factors in Queensland were supplied by the Australian Bureau of Meteorology, Queensland Department of Health, and Australian Bureau of Statistics, respectively. Three-stage spatiotemporal classification and regression tree (CART) models were developed to examine the association between social economic and weather factors and monthly incidence of cryptosporidiosis in Queensland, Australia. The spatiotemporal CART model was used for predicting the outbreak of cryptosporidiosis in Queensland, Australia.

**Results:**

The results of the classification tree model (with incidence rates defined as binary presence/absence) showed that there was an 87% chance of an occurrence of cryptosporidiosis in a local government area (LGA) if the socio-economic index for the area (SEIFA) exceeded 1021, while the results of regression tree model (based on non-zero incidence rates) show when SEIFA was between 892 and 945, and temperature exceeded 32°C, the relative risk (RR) of cryptosporidiosis was 3.9 (mean morbidity: 390.6/100,000, standard deviation (SD): 310.5), compared to monthly average incidence of cryptosporidiosis. When SEIFA was less than 892 the RR of cryptosporidiosis was 4.3 (mean morbidity: 426.8/100,000, SD: 319.2). A prediction map for the cryptosporidiosis outbreak was made according to the outputs of spatiotemporal CART models.

**Conclusions:**

The results of this study suggest that spatiotemporal CART models based on social economic and weather variables can be used for predicting the outbreak of cryptosporidiosis in Queensland, Australia.

## Background

Cryptosporidiosis is a diarrhoeal disease caused by microscopic parasites of the Cryptosporidium parvum [1]. The parasite is one of the most common causes of waterborne disease in Australia and globally and is found in drinking water and recreational water [2]. Cryptosporidiosis can also be transmitted via contaminated food, contact between people, or contact between people and animals. It is considered a drinking-waterborne disease because the largest outbreaks of cryptosporidiosis have been associated with contaminated drinking water. The individual risk factors for cryptosporidiosis in humans include drinking water from poorly treated public and private supplies, community swimming pools, day care centres and contact with farm animals. The seasonal occurrence of cryptosporidiosis has been cited as circumstantial evidence of a climatic link [3,4].

Spatiotemporal analyses of disease have played a major role in environmental epidemiology. Logistic regression, polyclass and generalised linear models have been widely used to study relationships between diseases and various environmental risk factors. However, these statistical techniques also perform relatively poorly with high dimensionality in spatiotemporal context [5-7]. For example, incidence rates, climate and social economic factors often involve high-order interactions and collinearities, which are often difficult to cope with using these models [8]. Moreover, infectious disease incidence data at finer spatial scales often have a preponderance of zero counts. In this case, the use of the conventionally employed standard statistical models may result in poor estimates and prediction [9-11]. An alternative approach for analysing such data with many zeros is to consider a suite of the spatiotemporal classification and regression tree (CART) models that take advantage of the natural categorisation of these types of populations into zero incidence, "normal" incidence and outbreaks. The CART models provide a non-parametric approach that can potentially better accommodancite these complex interactions since they avoid any assumption among the variables or homoscedasticity in variances [12]. The integrated use of spatial statistics and CARTs at a variety of spatial scales has provided new insights into ecology and environmental epidemiology [13-15].

The aims of this paper are to examine the potential impact of social economic and weather factors by LGAs on the incidence of cryptosporidiosis using spatiotemporal CART models and explore their potential as a predictive model for cryptosporidiosis in Queensland, Australia.

## Methods

### Study area

Queensland, located in the northeast of Australia, 10-28° south latitude and 138-153° east longitude, is the second largest state (after Western Australia) with the largest habitable area in Australia. It occupies the north-eastern quarter of the continent and covers approximately 1,727,000 km^2^, with 7,400 km of mainland coastline (9,800 km including islands). It has typically sub-tropical climate characteristics with average temperatures of 25°C in summer and 15°C in winter. Rainfall varies regionally and seasonally, and most of the state receives over 50% of its rainfall during summer. Average rainfall varies from less than 150 mm in the southwest region to more than 4,000 mm on the far northern coast. Queensland consists of 125 statistical local government areas (LGA), with populations ranging in size from 312 to 888,449. LGA are widely used by researchers in Australia because of the availability in many datasets.

### Data collection

We obtained the computerised data set on the notified cryptosporidiosis cases by LGA in Queensland for the period of 1^st ^January - 31^st ^December 2001 from the Queensland Department of Health. Weather and socio-economic index for areas (SEIFA) data were obtained for the same period from the Australian Bureau of Meteorology and the Australian Bureau of Statistics, respectively. SEIFA is a continuum of advantage (high values) to disadvantage (low values) and takes into account variables relating to education, occupation, wealth and living conditions. Australian Bureau of Statistics does not provide SEIFA data by temporarily (month-to-month). SEIFA variables vary spatially but rarely from month-to-month during one year period (ie., 2001) and each LGA had an observation for the period of this study. Weather data comprised monthly mean maximum temperature (°C) and monthly rainfall (mm), which was an interpolated climate surface with a 0.25 × 0.25° grid resolution (about 30 × 30 km) at the equator. Climate grid cells were calibrated using the GIS software package Vertical Map to extract average pixel values of the weather variables for each LGA.

### Spatial autocorrelation analysis

Moran's *I *spatial autocorrelation statistic was calculated to determine whether spatial clustering was a feature of cryptosporidiosis disease. Moran's *I *is defined by:

$$I(d)=\sum_{i}^{n}\sum_{j}^{n}w_{\text{ij}}(x_{i}-\bar{x}))(x_{i}-\bar{x}))/(S^{2}\sum_{i}^{n}\sum_{j}^{n}w_{\text{ij}})\text{ where }S^{2}=\frac{1}{n}\sum_{i}^{n}\left(x_{i}-\bar{x}\right))$$

*x*_*i *_*and x*_*j *_denote the observed value at location *i *and *j*, $\bar{x}$ is the average of the *x *values over the *n *locations, and *w*_*ij *_is the spatial weight measure [16]. Moran's *I *can be interpreted as follows: a value close to 0 indicates randomness, while a positive (negative) value indicates positive (negative) autocorrelation.

### Spatial empirical Bayes rates smoothing

We used a Bayesian random effects model to compute the spatial empirical Bayes rates smoothing and estimate the underlying distributions of incidence rates of cryptosporidiosis disease for each LGA and for Queensland overall. Spatial smoothing can be used to reduce random variation associated with small populations at each LGA. Spatial smoothing enables observations of gradients in cryptosporidiosis disease incidences that may not be apparent from the direct observation of raw incidences. Assume that the underlying rates for each LGA are drawn from a population of rates with prior distribution characterised by a mean *θ *and variance *φ*. The Bayesian posterior estimate for the underlying risk at the *i*th LGA is a weighted average of the raw rate *p*_*i *_and the prior, with weights inversely related to their variance. The equation is: ${\overset{\wedge }{\pi }}_{i}=w_{i}p_{i}+(1-w_{i})\theta$, where $w_{i}=\frac{\phi }{\phi +(\theta /P_{i})}$. *P*_*i *_is the population at risk in area *i *[16]. The smoothed incidences of cryptosporidiosis were computed from the total number of cryptosporidiosis cases in a spatial window divided by the total number of people at risk within the window. The spatial window was specified by a spatial weights file including each LGA and its neighbours. Thus the observed cryptosporidiosis rate for a LGA with a small population at risk will be adjusted considerably toward the average whereas for a larger LGA the rate will barely change.

The spatial empirical Bayes analyses were performed using GeoDa [16,17]. The locations of cryptosporidiosis cases by LGA were geo-coded to the digital base maps of localities using MapInfo [18], GeoDa [17] and Microsoft Access software.

#### Spatiotemporal CART models

Since monthly cryptosporidiosis count data at the LGA level have a high incidence of zero counts (about 85% in the dataset considered here), the analysis requires special care to account for the extra variation unaccounted for with a typical Poisson assumption. Moreover, these data typically here "outbreaks" characterised as very high incidences that can be difficult to model with a single parameter Poisson distribution. The mass of extra zeros can be thought of as two subgroups: LGA which due to some characteristic could not have cryptosporidiosis disease during the study period and LGA that could have cryptosporidiosis disease but this did not appear or was not reported. We considered a suite of three spatiotemporal CART models: 1) fitting a tree to data categorised as binary: incidence/no incidence; 2) fitting a tree to just the incidences (ignoring all of the zeros). The first two models are equivalent to a zero-inflated mixture model comprising some probability of a zero response (no incidence) and some probability of a (positive) truncated Poisson model [9]. Zero-inflated models are used when excess zeros result in a bimodal distribution which has a mixture of a mass of extra zeros and a mass that has a Poisson [10,19]. 3) fitting a tree for outbreak of cryptosporidiosis

#### CART 1: fitting a tree to data categorised as binary: incidence/no incidence

Classification trees are used to predict membership of cases or objects in the classes of a categorical dependent variable based on one or more exploratory variables [12]. A classification tree is built in accordance with a set of binary splitting rules that successively divide the data into subgroups with maximum homogeneity, where the latter is by some measure such as the change in the impurity [12]. A classification tree model was developed to explore possible relationships between socio-economic and weather factors and monthly cryptosporidiosis incidence. A dichotomous outcome variable was defined as whether or not one or more cryptosporidiosis cases occurred in the LGA in one month. The spatial CART model was thus described as: monthly cryptosporidiosis presence/absence ~ SEIFA + maximum temperature + rainfall + factor (season). The results are reported as the probability of occurrence of cryptosporidiosis in an LGA in a month.

#### CART 2: fitting a tree to positive incidences

Like a classification tree, a regression tree is built by binary recursive partitioning, but the response variable is continuous [12]. The splitting rules used in the algorithm are based on measures such as minimising the sum of the squared deviations from the mean in the two separate subgroups. The squared residuals minimisation algorithm is identical to a Gini splitting rule [12]. Positive monthly cryptosporidiosis incidence rates (1/100,000) by LGA were used as a continuous response variable in this model. The spatial CART model was thus described as: Monthly cryptosporidiosis rate ~ SEIFA + maximum temperature + rainfall + factor (season). The results are reported as the relative risk (RR) of positive incidence of cryptosporidiosis, compared to average incidence rate of cryptosporidiosis. RR = (expected incidence - mean of incidence)/mean of incidence.

#### CART3: fitting a tree to outbreaks of cryptosporidiosis

We defined an outbreak if the incidence rate (without zeros) in any month exceeded the third quartile of the incidence rates per LGA in Queensland during the 12 months of 2001. The spatial CART model was thus described as: monthly cryptosporidiosis outbreak/non-outbreak ~ SEIFA + maximum temperature + rainfall + factor (season). The results are reported as the probability of occurrence of an outbreak cryptosporidiosis in a month. A map of predicted probabilities was developed using the outputs of the CART model.

Each CART analysis consisted of three basic steps. Firstly, a preliminary tree was grown by recursive data partitioning. Secondly, nested trees were formed by reducing the number of nodes in the tree (pruning). Thirdly, 10-fold cross-validation was used to address over-fitting and to identify the optimal tree with respect to its predictive ability. A minimum node deviance of 25% of the total deviance was used to prune the trees. To control for the impact of seasonality, we decomposed the cryptosporidiosis incidence into four seasonal categories (coded as Spring: September-October-November; Summer: December-January-February; Autumn: March-April-May; Winter: June-July-August) [20,21]. Finally, we validated the model and tested the residual mean deviance and misclassification rates (predicted number of months without cryptosporidiosis occurrence/total number of months). The statistical analysis was conducted using the S-plus software package [22].

For each CART model, the predicted probabilities or relative risks can be presented as a map. A map of predicted probabilities of an outbreak was developed using the outputs of the CART 3 model.

## Results

### Descriptive analysis

**Figure 1:**
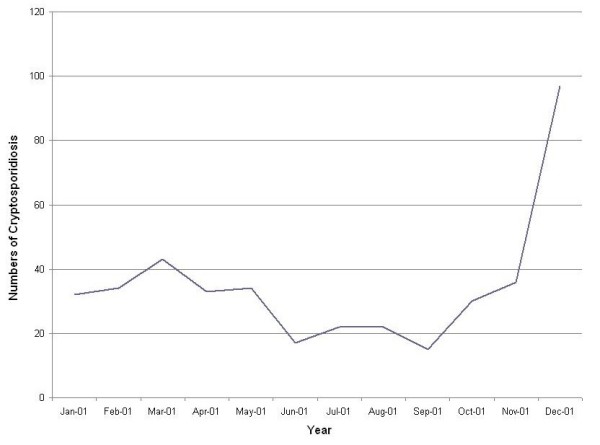
**The cryptosporidiosis counts between January 2001 and December 2001 in Queensland, Australia**.

Table 1 shows the summary statistics for each variable. The monthly mean incidence rate of cryptosporidiosis, maximum temperature, rainfall and SEIFA were 15.11, 28.17°C, 59.64 mm and 934.17, respectively. Figure 1 depicts the variation over time in cryptosporidiosis between January 2001 and December 2001. The curves indicate a seasonal pattern. About 45% of cryptosporidiosis cases occurred in children under 4 year old in 2001.

**Table 1:** Descriptive statistics of monthly cryptosporidiosis and social economic and weather variables by LGA in Queensland*

	Mean	SD	Minimum	Q1	Median	Q3	Maximum
Cryptosporidiosis incidence (1/100,000)	15.11	69.07	0.00	0.00	0.00	1161.67	1332
Maximum temperature (°C)	28.17	4.61	16.54	24.55	28.48	31.49	40.11
Total rainfall (mm)	59.64	80.18	0.00	14.44	40.26	72.70	916.60
SEIFA	934.17	43.17	831.36	907.28	928.56	963.04	1059.84

* Q1: first quartile value; Q3: third quartile value

### Bivariate analyses

**Figure 2:**
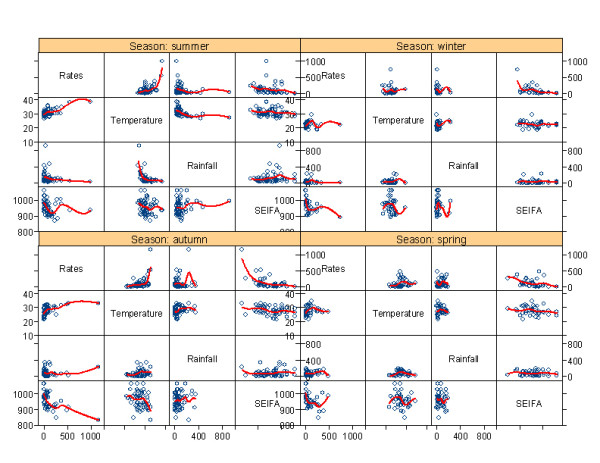
**Pairwise scatterplot (with regression line) of cryptosporidiosis without zero incidences and explanatory variables**.

**Figure 3:**
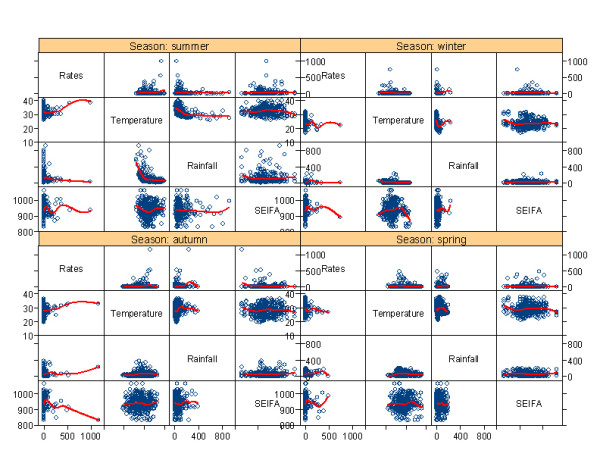
**Pairwise scatterplot (with regression line) of cryptosporidiosis with zero incidences and explanatory variables**.

Table 2 shows the linear associations between the cryptosporidiosis incidence and temperature, rainfall and SEIFA. It also summarises the bivariate linear relationships between all the independent variables. For the database without zero incidences, SEIFA (r = -0.535, p = 0.000) and temperature (r = 0.349, p = 0.000) were significantly associated with cryptosporidiosis incidence. For the full database including zero incidences, rainfall (r = 0.152, p = 0.000) and SEIFA (r = 0.192, p = 0.000) were significantly linearly associated with cryptosporidiosis incidence. Rainfall was also statistically associated with temperature for the database without zero incidence (r = 0.346, p = 0.000) and for the full database (r = 0.378, p = 0.000). The pairwise scatter-plot with spline regression line depicts the relationships between all the variables by season in Figure 2. As suggested by this figure, the incidence rates were associated with the SEIFA and temperature for the database without zero incidences in summer and autumn. However, the incidence rates were not associated with the SEIFA and temperature for the full database in any season (Figure 3).

**Table 2:** Matrix of correlation coefficients between cryptosporidiosis and social economic and weather factors*

	Incidences	Temperature	Rainfall
Temperature (°C)	0.349† (-0.033)		
Rainfall (mm)	-0.008 (0.152†)	0.346† (0.378†)	
SEIFA	-0.535† (0.192†)	-0.213† (0.024)	0.018 -0.035

*:Coefficients for data without zero incidence, and for data with zero incidence in brackets; †:p < 0.01

There was a significant positive spatial autocorrelation of cryptosporidiosis incidence (including zero incidences) with a Moran's *I *statistic of 0.213 (p = 0.015).

### Spatial distribution empirical Bayes rates smoothing

**Figure 4:**
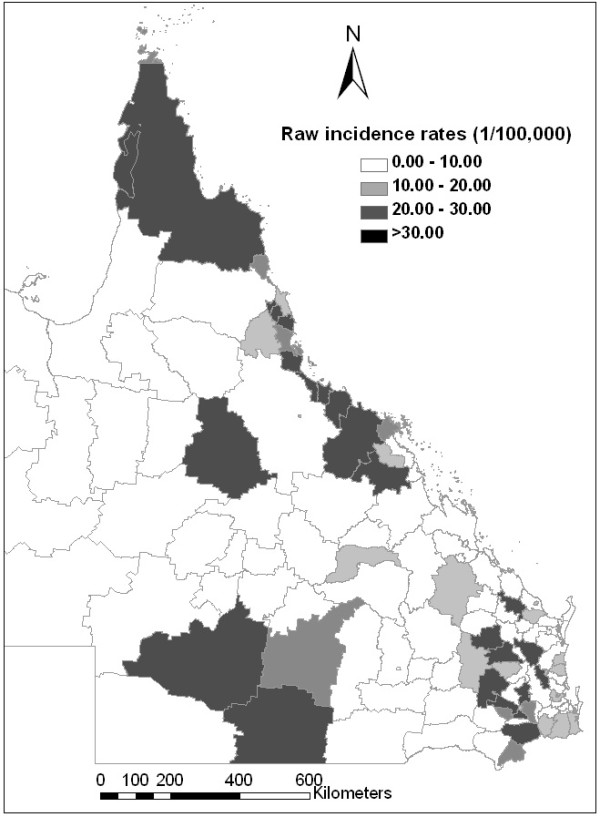
**Choropleth map of notified incidence rates of cryptosporidiosis**.

**Figure 5:**
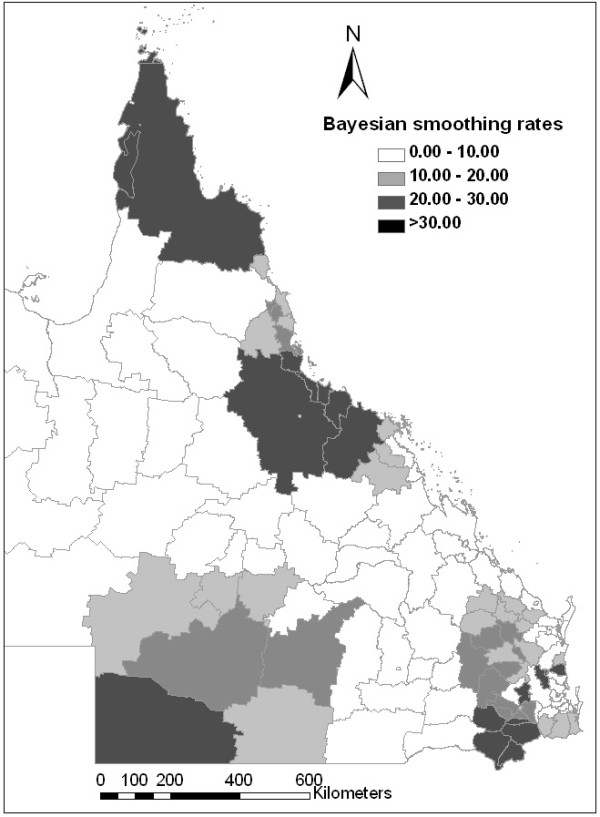
**Choropleth map of spatial empirical Bayesian smoothed incidence rates of cryptosporidiosis**.

Figure 4 depicts the geographic distribution of the notified cryptosporidiosis incidences in Queensland during 2001. The figure confirms that the risk for cryptosporidiosis varied with geographical location. The Bayesian posterior estimates of the cryptosporidiosis rates for each LGA are depicted in Figure 5. The spatial empirical Bayes analysis showed that the cryptosporidiosis infection activity was primarily concentrated in the north, southeast and southwest of Queensland, Australia.

### CART 1 model

**Figure 6:**
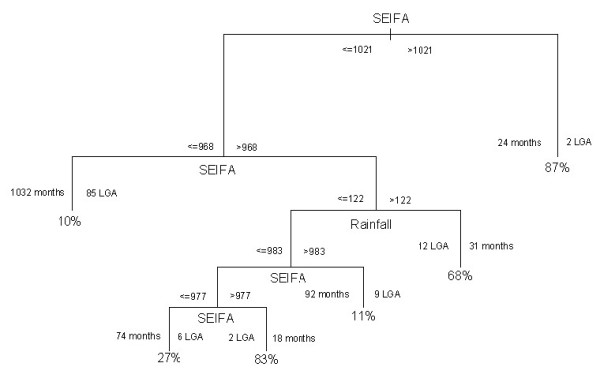
Results of CART model of probability of presence of cryptosporidiosis,

Figure 6 represents the final CART model for presence/absence of cryptosporidiosis. This figure indicates that presence of cryptosporidiosis was predominantly explained by SEIFA, with an 87% chance of an occurrence if the SEIFA was in excess of 1021 (2 LGA, 24 months). The validation analyses indicate that the misclassification error rate was13% and residual mean deviance was 0.72.

### CART 2 model

**Figure 7:**
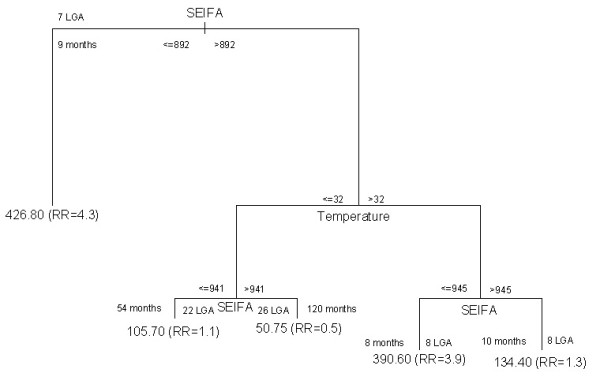
**Results of CART model of expected incidence of cryptosporidiosis**.

Figure 7 represents the final CART model for positive incidence of cryptosporidiosis in a month, by LGA. The results indicate that when the SEIFA was between 892 and 945, and temperature was over 32°C (8 LGA, 8 months), the relative risk (RR) of cryptosporidiosis rose to 3.9 (mean morbidity: 390.6/100,000, standard deviation (SD): 310.5), compared to monthly average incidence of cryptosporidiosis. When the SEIFA was less than 892 (7 LGA, 9 months) the RR of cryptosporidiosis was 4.3 (mean morbidity: 426.8/100,000, SD: 329.2), compared to the same baseline. No further splits were found to substantially improve the homogeneity of the subgroup outcome of non-zero incidence. The validation analyses indicate that the residual plots appear reasonable well.

### CART 3 model

**Figure 8:**
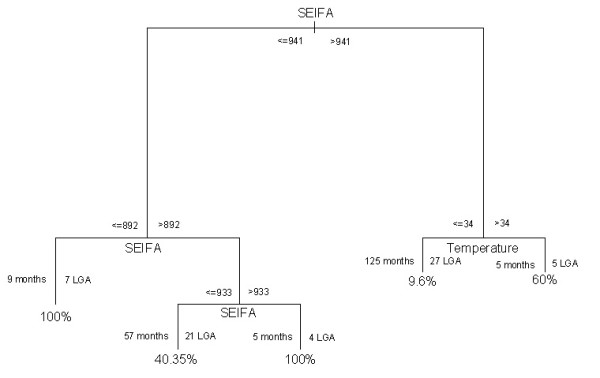
**Results of CART model of probability of an outbreak**.

**Figure 9:**
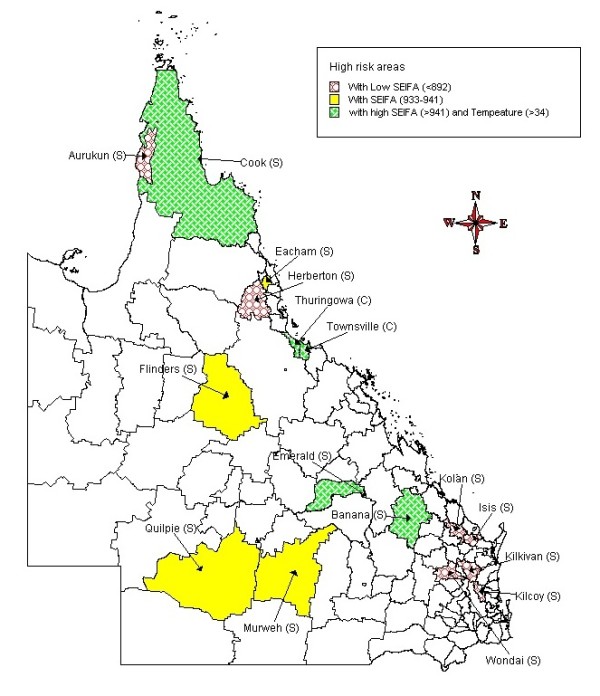
**Risk map based on spatiotemporal CART model**.

Figure 8 represents the final CART model which indicates that an outbreak of cryptosporidiosis, defined as exceeding the third quartile of the incidence rates, was best explained by SEIFA. The analysis indicates that there was a 100% chance for an outbreak of cryptosporidiosis if the SEIFA was less than 892 (7 LGA, 9 months) or the SEIFA was between 933 and 941 (4 LGA, 5 months) during an epidemic period. There was a 60% chance of an outbreak of cryptosporidiosis if the SEIFA was greater than 941 and temperature was greater than 34°C (5 LGA, 5 months) during an epidemic period. Figure 9 shows a high risk map of predicted monthly cryptosporidiosis incidence rates in Queensland based on the CART models, which took into account local variation (ie., SEIFA, temperature and season) from the model prediction. The validation analyses indicate that the misclassification error rate was18% and residual mean deviance was 0.73.

These results of validation analysis reveal that the three CART models had reasonable accuracy, and its utility in research needs to be further explored.

## Discussion

The results of this study indicate that there was remarkable variation in the spatial distribution of cryptosporidiosis and suggest that temperature and SEIFA are statistically associated with the probability of occurrence and the magnitude of the incidence of the disease in Queensland, either directly or through other unmeasured variables.

Cryptosporidiosis is one of the common waterborne diseases globally. Therefore, from a public health perspective, there is a need to control and prevent this disease. There is evidence that education and behaviour might be direct risk factors for cryptosporidiosis infection [23]. People with a lower SEIFA level often have poor health knowledge about disease transmission and personal protection from water-borne diseases. Thus, it is important to educate people in lower SEIFA areas about how to avoid contracting this infection especially in epidemic periods.

*Cryptosporidium parvum *thrive in warm waters of moderate salinity. Thus, with changing weather patterns, the geographic range of cryptosporidiosis may also change. For example, severe weather events appear to be correlated with outbreaks of cryptosporidiosis in hot summers in Brisbane and South Sydney, Australia [24,25]. In the summer and autumn the cryptosporidiosis rate was positively associated with temperatures in the current and previous month in New Zealand [26]. The autumn cryptosporidiosis peak has been linked to increased recreational water use, swimming and outdoor activities [27]. Our research shows that high temperature (>32°C) has a significantly effect on cryptosporidiosis incidence in LGAs with high SEFIA values. This could be explained by the observation that people in these higher SEIFA classes may be more likely to undertake recreational activities such as camping and swimming in high temperature which may increase the chance of *cryptosporidium *[28]. SEIFA allows ranking of regions/areas, providing a method of determining the level of social and economic well-being in each region. Although SEIFA is a useful overall index, two areas may have a similar SEIFA score for very different reasons. Thus, it may be important to require additional detailed information (eg., education and income etc) for particular analyses. Our previous research has shown that the proportion of residents with low educational attainment has a positive and significant association with cryptosporidiosis, especially in the regions with high SEIFA value [15].

Three-stage spatiotemporal CART models are based on simple splits of the data and thus do not require the assumptions such as the existence of linear regression among the variables [12]. The visualized tree structure of the CART model makes the analysis results easier to understand and explain. The structure of the CART model is a set of nodes from the top to the bottom, in which the terminal nodes show the specific pattern features of the subpopulations (eg, the number of SLA, mean, risk, and probability etc) [15].

The suite of spatiotemporal CART models proposed have provide a comprehensive approach to modelling count data that have many zero counts and few very large counts. Because the ordinary CART do not take into account the extra variation, the test of regression parameters often fails to control Type I error rate. To overcome this problem, we developed three stage CART models. Our study shows that the three CART models can be applied in count data with extra zeros. The models can determine which predictors affect the probability of being an incidence of cryptosporidiosis, which predictors affect how positive incidence rates and which predictors affect the probability of an outbreak of cryptosporidiosis. The three models use the same social economic and weather predictors, but allow for different interactions and estimates of relative importance of the variables in describing the difference outcomes. Hence the predictors appear vastly different effects on the three models. They may not be the same predictors for the three models, or they could even have opposite effects on the three outcomes. It is thus advantageous to consider the set of models together when assessing the association between cryptosporidiosis disease and social economic and weather factors in the model.

The models proposed here can be extended in a number of lags. A lag of 0 month was considered because of biological plausibility (i.e., the incubation period for cryptosporidiosis ranges from 2 to 12 days). Longer lags (e.g., ≥ 1 month) were found to be less important in this study.

This study has four major strengths. Firstly, to our knowledge, this is the first spatiotemporal study examining the spatial variation of cryptosporidiosis disease and temperature, rainfall and SEIFA at a LGA level. Secondly, three spatiotemporal CART models were used to identify overall patterns of cryptosporidiosis transmission. Thirdly, detailed information on social economic and weather factors (temperature, rainfall and SEIFA) by LGA was incorporated in the statistical models, which may be helpful for further ecological research and development of early warning system. Finally, a map of predicted occurrence of cryptosporidiosis from this study may have important implications for public health decision-making in identifying high-risk communities to target for control and prevent cryptosporidiosis infection.

Limitations of this study should also be acknowledged. Firstly, the study suffers from the usual problems of an ecological design [29]. Further researches need to take into account temporal change in long term period (ie., more than 5 years) during the ecological studies at fine spatial scales (ie., postal areas). Secondly, the mechanism and pathways underlying the association of cryptosporidiosis with SEIFA and temperature remain unclear [4]. In this study, only the SEIFA, temperature and rainfall were considered. Other factors (eg., water reservoirs or large farms) may also be directly or indirectly related to cryptosporidiosis transmission. However these data were unavailable in this study. Finally, under-reporting is likely to occur when people infected by cryptosporidiosis have sub-clinical conditions and/or did not seek to see a doctor because they knew there is no effective treatment for this disease.

The identification of disease high risk areas provided the chance to explore social economic and weather factors that may be responsible for developing early warning systems of cryptosporidiosis. Early outbreak detection provides the chance to implement control measures and education campaigns. The effectiveness of public health interventions can be improved using spatiotemporal models to identify and monitor hot spots of cryptosporidiosis and identify major risk factors and the risk areas of cryptosporidiosis, and then targeting education campaigns and disease control activities at specific areas.

## Conclusions

The findings of this study indicate that spatiotemporal CART models based on social, economic and weather variables can be used for predicting the outbreak of cryptosporidiosis in Queensland, Australia, and the research approach developed in this study may have wide applications in the surveillance and risk management of infectious diseases.

## Competing interests

The authors declare that they have no competing interests.

## Authors' contributions

WH was the principal investigator and was responsible for the design of the study, collection of the data, development of analytical protocol, interpretation of the results and writing of the paper. KM and ST contributed to the design of the study, the interpretation of results and writing of the paper. All authors read and approved the final manuscript.

## Pre-publication history

The pre-publication history for this paper can be accessed here:

http://www.biomedcentral.com/1471-2334/10/311/prepub
